# Cherish Your Exceptions, But Also Your Doubts

**DOI:** 10.1089/rej.2019.2178

**Published:** 2019-02-28

**Authors:** Aubrey D.N.J. de Grey

**Affiliations:** SENS Research Foundation, Mountain View, California.

“Treasure your exceptions!”

—William Bateson

The article following this editorial argues for a hypothesis that has already engendered substantial turmoil within the gerontological community since its essence was revealed to the media (by another researcher) a couple of months ago. Is it a new hallmark of aging? No. Is it a new way to combat an existing hallmark? No again. In fact it is a challenge to arguably the single most famous “fact” in gerontology (at least when fame is measured among the general public rather than professionals): the world record of human longevity. According to the article, Jeanne Calment probably died not in 1997 but in 1934. I will not summarize the article here; after all, you only need to turn the page. Instead I will offer some thoughts on what this development can teach us about how to do our jobs as scientists engaged in humanity's most important crusade, the elimination of aging.

Bateson's principle is timeless for scientists: it is on a par with Occam's Razor as regards choosing wisely what experiment to do next. When a data point looks wrong, that is because it does not fit your world view, your null hypothesis—and that can mean one of only two things: your data point is a product of experimental or observational error, or your null hypothesis is incorrect. Thus, designing a new experiment focused on that data point is the way to determine which of those two is the case, so it is a really good idea.

Why did Bateson need to point this out? Interestingly, not for today's reason. He did so because it is mighty tempting to assume the former alternative, that is, to ignore an anomalous data point on the basis that something can surely explain it away, even though one has not actually identified that something. But today's topic concerns the opposite tendency: having convinced oneself that an ostensibly anomalous data point is valid, to become invested in that belief at the expense of new information that might motivate the opposite conclusion. A saddening number of colleagues, including some from whom I would honestly have expected greater caution, have already been guilty of this error regarding the Calment article, in the aftermath of the prepublication publicity noted previously.

Some of you may recall that I complained in a previous editorial^1^ about the way in which available information on supercentenarians is disseminated. The main focus of my complaint was precisely the thing that is highlighted here: that experts must not play fast and loose with uncertainty. This applies across the board: actual examples include cases such as Toshi Horiya that simply do not appear in official lists because their date of death is ambiguous by a single day, as well as cases such as Lucy Hannah that do appear even though decisive evidence came to light years ago that they did not live nearly as long as was originally believed.

But this is a much more general problem: it applies across the whole of science, and arguably especially so for gerontologists, given the humanitarian importance of our work. The blame ultimately lies with the career structure of science, which exerts immense pressure to stick to one's guns concerning any opinion that one has publicly stated in the past. That pressure must be resisted. It is doubtless also fueled by personal egos, but that is no excuse.

I am often asked for my view of why the San Francisco Bay area has acquired, and has maintained for decades, the status of world leader in technological innovation (which, of course, played a large part in why it has become my home). My answer is that people here have a completely different attitude toward other people's failures than exists elsewhere. In the rest of the world, when you fail, people default to the assumption that you are not very good, so their enthusiasm to help you to try again is diminished. In the Bay, when you fail, the default assumption is that you are courageous enough to have attempted something really hard, so their enthusiasm to help you to try again is increased! Of course people are sometimes right and sometimes wrong in both cases—but what matters is the leverage, the positive feedback loop. When the Bay area attitude is wrong, a little money gets lost. When the rest-of-the-world attitude is wrong, a little money gets saved that would have multiplied. When the rest-of-the-world attitude is right, a little money gets saved that would have been lost. But when the Bay area attitude is right, someone truly courageous gets to succeed at the second or fourth attempt, and when that kind of person succeeds, they tend to succeed in spades (precisely because they are the sort of persons who are willing to try and try and try again), and become the people who will help the next round of courageous people.

What does that have to do with my theme? It is the exact same thing. In science today, it is not okay to have been wrong—and that is a disaster. Science would progress so much faster if scientists were applauded, rather than downgraded, for changing their views in the light of new data. This is not a new problem; indeed, in one of my first editorials my quotation was the memorable observation of Bateson's contemporary Max Planck that “science advances funeral by funeral.” But still the problem persists. We need somewhere (ideally everywhere, of course) that is for science what the Bay area is for technology. And we need it now.
